# From IB2 to IIIB locally advanced cervical cancers: report of a ten-year experience

**DOI:** 10.1186/s13014-018-0963-8

**Published:** 2018-02-02

**Authors:** Sophie Espenel, Max-Adrien Garcia, Jane-Chloé Trone, Elodie Guillaume, Annabelle Harris, Amel Rehailia-Blanchard, Ming Yuan He, Sarra Ouni, Alexis Vallard, Chloé Rancoule, Majed Ben Mrad, Céline Chaleur, Guy De Laroche, Jean-Baptiste Guy, Pablo Moreno-Acosta, Nicolas Magné

**Affiliations:** 1 Radiotherapy Department, Lucien Neuwirth Cancer Institute, 108 bis avenue Albert Raimond, BP60008, 42271 Saint-Priest-en-Jarez cedex, France; 2 Public Health Department, Lucien Neuwirth Cancer Institute, 108 bis avenue Albert Raimond, BP60008, 42271 Saint-Priest-en-Jarez cedex, France; 3 Chelsea and Westminster Hospital, 369 Fulham Road, London, SW10 9NH UK; 4 Obstetrics and Gynecology Department, Saint Etienne University Hospital Medical Center, avenue Albert Raimond, BP60008, 42271 Saint-Priest-en-Jarez cedex, France; 5 Research Group in Radiobiology Clinical, Molecular and Cellular, National Cancer Institute, Bogotá, Colombia; 6 Research Group in Cancer Biology, National Cancer Institute, Bogotá, Colombia

**Keywords:** Locally advanced cervical cancer, Standard care, Daily clinical practice, Radiotherapy, Chemotherapy, Brachytherapy

## Abstract

**Background:**

Despite screening campaigns, cervical cancers remain among the most prevalent malignancies and carry significant mortality, especially in developing countries. Most studies report outcomes of patients receiving the usual standard of care. It is possible that these selected patients may not correctly represent patients in a real-world setting, which may be a limitation in interpreting outcomes. This study was undertaken to identify prognostic factors, management strategies and outcomes of locally advanced cervical cancers (LACC) treated in daily clinical practice.

**Methods:**

Medical files of all consecutive patients treated with curative intent for LACC in a French Cancer Care Center between 2004 and 2014 were reviewed retrospectively.

**Results:**

Ninety-four patients were identified. Performance status was ≥ 2 in 10.6%. Median age at diagnosis was 63.0. Based on the International Federation of Gynecology and Obstetrics classification, tumours were classified as follows: 10.6% IB2, 22.3% IIA, 51.0% IIB, 4.3% IIIA and 11.7% IIIB. Pelvic lymph nodes were involved in 34.0% of cases. Radiotherapy was delivered for all patients. Radiotherapy technique was intensity modulated radiation therapy or volumetric modulated arc therapy in 39.4% of cases. A concurrent cisplatin chemotherapy was delivered in 68.1% of patients. Brachytherapy was performed in 77.7% of cases. The recommended standard care (concurrent chemoradiotherapy with at least five chemotherapy cycles during radiotherapy, followed by brachytherapy) was delivered in 43.6%. The median overall treatment time was 56 days. Complete tumour sterilisation was achieved in 55.2% of cases. Mean follow-up was 54.3 months. Local recurrence rate was 18.1%. Five-year overall survival was 61.9% (95% Confident Interval (CI) = 52.3–73.2) and five-year disease-specific survival was 68.5% (95% CI = 59.2–79.2). Poor performance status, lymph nodes metastasis and absence of concurrent chemotherapy were identified as poor prognostic factors in multivariate analysis.

**Conclusions:**

Less than 50% of patients received the standard care. Because LACC patients and disease are heterogeneous, treatment tailoring appears to be common in current clinical practice. However, guidelines for tailoring management are not currently available. More data about real-world settings are required in order to to optimise clinical trials’ aims and designs, and make them translatable in daily clinical practice.

**Trial registration:**

retrospectively registered.

## Background

Despite screening campaigns, cervical cancers remain among the most prevalent malignancies and carry significant mortality, especially in developing countries [1]. Each year, more than 500,000 new cases and 260,000 deaths are reported worldwide. Many patients are diagnosed with locally advanced stages. A concomitant chemoradiotherapy (CCRT), consisting of cisplatin based chemotherapy alongside external beam radiotherapy (EBRT), followed by brachytherapy is considered to be the standard care [2, 3]. A completion surgery may be performed, but the benefits are uncertain [3–5]. Previous literature has reported a 5-year overall survival (OS) ranging from 39.2% to 80% in locally advanced cervical cancer (LACC) patients (Table 1) [5–9]. Many prognostic factors were identified- patient characteristics (age [9–11], performance status [11], comorbidities [12, 13]), disease characteristics (stage [6, 9, 11, 14], tumor size [11], lymph nodes metastasis [5, 6, 11], histological type and differentiation [11, 15]) and treatment characteristics (overall treatment time (OTT) [16], concurrent chemotherapy [6, 15, 17–19] and brachytherapy boost [2, 6, 20, 21]). However, 30 to 40% of patients with similar recognised prognostic factors seem to respond to treatments differently [22]. Biological theories are a current area of research, especially the analysis of the subpopulation of radioresistant tumour cells. The impact of human papillomavirus (HPV) status and variants on prognosis has been identified [22–25], and may lead to the development of antiviral anticancer treatments [26]. Another hypothesis may explain outcomes heterogeneities between publications and daily clinical practice. Most studies assessed prognostic factors and reported outcomes of selected patients receiving the standard care. It is possible that these selected patients may not correctly represent patients in a real-world setting. Therefore, it is of paramount importance to identify prognostic factors in such patients with LACC. This may play a crucial role in tailoring anticancer treatments. The aim of this study was to identify prognostic factors, management strategies and outcomes of LACC treated in daily clinical practice.

**Table 1:** OS’s comparisons in recent studies

Study	Centre	Number of patients	Median age (years)	FIGO IB2 (%)	FIGO II (%)	FIGO III (%)	FIGO IV (%)	Pelvic LNI (%)	Lombo aortic LNI (%)	Median/Mean follow-up (months)	DFS % (CI 95%)	OS % (CI 95%)	DSS % (CI 95%)
Present study	Saint Etienne, France	94	63	10.6	73.3	16	0	34.1	0	43.9/54.3	56.7 (46.9–68.5)	61.9 (52.3–73.2)	68.5 (59.2–79.2)
Sturdza, [6]	International cohort	731	53	16.8	55.9	22.9	3.1	40.5		43	65	–	73
Touboul, [5]	Villejuif, France	150	47	32	61	7	0.7	32.7	0.7	−/43.2	66 (57–74)	71 (61–78)	–
Haie-Meder, [8]	Villejuif, France	84	46.5	23.8	44	26.2	6	36.9		53/−	57* (43–69)	52* (40–64)	–
Han, [21]	Toronto, Canada	7359	55 (mean)	8.2	52.5	35.8	3.5	–	–	40.8/−	–	58.2* (with brachytherapy), 46.2* (without)	64.3* (with brachytherapy), 51.5* (without)
Magne, [14]	Villejuif, France	113	76	3.5 (31% IA and IB1)	45,1	16,8	3.6	14.2	6.2	37.2/−	81** (72–88)	88.6** (77–92)	–
Gouy, [7]	Villejuif, France	237	46	33	55	10	2	21	–	30/−	71** (63–78)	78** (64–88)	–
Song, [28]	Chicago, US	113	49	19	57	24	0	17	0	26/−	58** (49–67)	66** (57–75)	74** (66–82)
Chargari, [37]	Villejuif, France	45	50	31.1	51	13.3	4.4	51.1	8.9	26/26.7	73***	78***	–

Pathological lymph nodes were defined as lymph nodes > 1 cm in size, loss of oval shape on imaging, or positive on PET-CT imaging

Survivals are given at 5-year, 4-year (*), 3-year (**) or 2-year (***)

Abbreviations: *FIGO* International Federation of Gynecology and Obstetrics, *LNI* lymph node involvement, *DFS* Disease-Free Survival, *OS* Overall Survival, *DSS* Disease-Specific Survival, *PET-CT* Positron Emission Tomography-Computed Tomography

## Methods

A retrospective study was conducted at the Lucien Neuwirth Comprehensive Cancer Care Center (Saint-Priest-en-Jarez, France). The database was declared to the French Commission of Informatics and Freedom. The study was conducted in compliance with ethical standards and with the 1964 Helsinki Declaration.

### Patient population

Medical records of all consecutive patients treated with curative intent for a LACC between January 2004 and December 2014 were retrospectively reviewed by a single investigator. Clinical and dosimetric data were collected. LACC was defined as stages IB2 to IIIB according to the 2009 International Federation of Gynecology and Obstetrics (FIGO) classification, regardless of the lymph node status. An ecological index of social deprivation was used to estimate patients’ socio-economic status (the French version of the European Deprivation Index [EDI]). Patients were classified into quintiles according to their degree of deprivation, from 1 (least deprived) to 5 (most deprived). Alive patients were contacted in 2017 to obtain the most recent follow-up data.

### Work-up and treatment definition

#### Work up

Pelvic magnetic resonance imaging (MRI) has been available since 2004, and Positron Emission Tomography-Computed Tomography (PET-CT) since 2009. For patients included in ERRICC clinical trial (diagnostic performance of 18F- Fluodesoxyglucose -PET and diffusion-weighted MRI in the assessment of stage IB to IIB2 cervical squamous-cell carcinoma response to concomitant radiochemotherapy and brachytherapy, NCT01663753), para-aortic lymph node dissection was performed if no para-aortic involvement was highlighted on the initial PET-CT.

#### Treatment

A conventionally fractionated radiation scheme (1.8 to 2Gy per fraction) was performed by external beam radiotherapy (EBRT) on the pelvis +/− para-aortic lymph nodes (*ei* para-aortic lymph nodes involvement according to PET-CT or dissection), to a total dose of at least 45Gy. Pathologic lymph nodes received a higher dose. The clinical target volume (CTV) consisted of the gross tumour volume (GTV), uterus, parametria, vagina at least 2 cm below the GTV and the pelvic lymph node regions. The planning target volume (PTV) consisted of the CTV with additional 1 to 2 cm margins. Doses were prescribed and recorded in compliance with the international standard. All treatment plans were optimised according to dose limits for organs at risk. Three-dimensional conformal radiotherapy (3D–CRT) was already available before 2004. For 3D–CRT, dose-at-a-point prescription was performed, according to the International Commission on Radiation Units and Measurements (ICRU)-50 and − 62. The ICRU point should be located in the central part of the target volume (centroid), and if possible at the point of intersection of the beams. In addition, the dose received in each point of the target volume should be between 95% and 107% of the prescribed dose.

Intensity modulated radiotherapy (IMRT) was used since 2008, and volumetric modulated arc therapy (VMAT) since 2011. For IMRT and VMAT, a dose-volume prescription (corresponding to an isodose) was performed according to the ICRU-83. For each target volume, the median dose (D50%) and the doses received by 2%, 90%, 95% and 98% of the target volume (respectively D2%, D90%, D95%, D98%) were reported. Treatment was delivered with a full bladder, and an empty rectum. Cone-beam computed tomography (CBCT) was performed on day one, day two, day three and then twice a week during IMRT or VMAT treatment. Patients underwent concurrent chemotherapy except in cases of contra-indication, incompatible age or refusal. Concurrent chemotherapy consisted of weekly cisplatin 40 mg/m^2^, or weekly carboplatin area under the curve (AUC) 2 where cisplatin was contra-indicated. This was followed by intracavitary brachytherapy, using a ring and tandem applicator (Nucletron, Sweden from 2004 to 2012; Varian, USA since 2012). Pulsed-dose rates (PDR) with an Iridium-192 stepping source were used. Continuous hourly pulses were delivered 24 h/day. Treatment planning was based on computed tomography-scan (CT-scan) based dosimetry. Doses were prescribed at point A, according to the American Brachytherapy Society guidelines, and were ≥60Gy. Dose constraints to the bladder and to the rectum were reported according to the ICRU-38 recommendations. No concurrent chemotherapy was delivered during brachytherapy. Finally, colpohysterectomy with bilateral adnexectomy and pelvic lymphadenectomy (CHL) could be performed following a multidisciplinary team decision. Because no recent prospective clinical trial has demonstrated equivalent outcomes with or without completion surgery, CHL was performed for each patient, except in cases of inclusion in ERRICC clinical trial, contra-indication or refusal.

### Statistics and results

Local response to treatment was assessed through histological examination. Efficacy and toxicity of treatment were assessed every three to six months during the subsequent five years, based on physical examination. Biopsies were systematically performed in cases where there was suspicion of tumour recurrence. Local failure was defined as any recurrence in the cervix, parametria, vagina or uterus. Survival rates were calculated from diagnosis to the occurrence of the studied event. The Kaplan Meier method was used to obtain curves of overall survival (OS), disease-specific survival (DSS) and disease-free survival (DFS). 5-year OS, 5-year DSS and 5-year DFS were given with their 95% confident interval (95% CI). Median values were given with their interquartile range (IQR). All *p* values were nominal without adjustment for multiple testing. Significance was defined by *p* < 0.05. The multivariate analysis was performed using a Cox multivariate analysis based on the significant factors in univariate analysis (log rank test). The multivariate model was refined using the Akaike Information Criterion (AIC). Statistical analyses were processed with R-3·2·2 (R Core Team. R Foundation for Statistical Computing, Vienna, Austria).

## Results

### *Patient characteristics* (Table 2)

A total of 94 patients fulfilling the inclusion criteria were identified. Median age at diagnosis was 63.0 (IQR = 51.3–75.0), with 46.8% over 65 and 39.4% over 70. Performance status was 2 for 10.6% of the patients. Charlson combined age-comorbidity index was 0 for 9 patients (9.6%), 1–2 for 29 patients (30.9%), 3–4 for 31 patients (33.0%) and ≥ 5 for 25 patients (26.6%). The EDI revealed that 53 patients (56.4%) appeared to have social deprivation (quartile 4 and 5). Tumours were classified as follows: 10 IB2 stages (10.6%), 21 IIA stages (22.3%), 48 IIB stages (51.0%), 4 IIIA stages (4.3%) and 11 IIIB stages (11.7%). Pelvic lymph nodes were involved in 34.0% of patients. The two main histologic types were squamous cell carcinoma (76.6%) and adenocarcinoma (14.9%). A non squamous cell carcinoma was reported in 32.4% of patients > 70 years. The mean pre-radiation haemoglobin level was 12.9 g/dL.

**Table 2:** Patient characteristics (*n* = 94)

Characteristics	Whole set of patients (*n* = 94)	Complete CCRT (*n* = 41)	Incomplete CCRT (*n* = 53)	*p*-value
Age, years, median (IQR)	63.0 (51.3–75.0)	59.1 (50.7–73.4)	65.3 (54.4–76.0)	NS
PS, n (%)				
PS ≤ 1	83 (88.3)	24 (58.5)	20 (37.7)	0.03^a^
PS ≥ 2	10 (10.6)	16 (39.0)	33 (62.3)	
UK	1 (1.1)	1 (2.4)	0 (0)	
Charlson combined age-comorbidity index, n (%)				
0	9 (9.6)	4 (9.8)	5 (9.4)	NS
1–2	29 (30.9)	16 (39.0)	13 (24.5)	
3–4	31 (33.0)	13 (31.7)	18 (34.0)	
≥ 5	25 (26.6)	8 (19.5)	17 (32.1)	
Quintile EDI				
1	8 (8.5)	4 (9.8)	4 (7.5)	NS
2	15 (16.0)	8 (19.5)	7 (13.2)	
3	11 (11.7)	2 (4.9)	9 (17.0)	
4	24 (25.5)	14 (34.1)	10 (18.9)	
5	29 (30.9)	11 (26.8)	18 (34.0)	
UK	7 (7.4)	2 (4.9)	5 (9.4)	
Histological type, n (%)				
Squamous cell carcinoma	72 (76.6)	33 (80.5)	39 (73.6)	NS
Adenocarcinoma	14 (14.9)	6 (14.6)	8 (15.1)	
Other	8 (8.6)	2 (4.9)	6 (11.3)	
Differentiation grade, n (%)				
High	24 (25.5)	12 (29.3)	12 (22.6)	NS
Moderate	26 (27.7)	12 (29.3)	14 (26.4)	
Low	32 (34.0)	12 (29.3)	20 (37.7)	
UK	12 (12.8)	5 (12.2)	7 (13.2)	
FIGO Stage, n (%)				
IB2	10 (10.6)	5 (12.2)	5 (9.4)	NS
IIA	21 (22.3)	15 (36.6)	16 (30.2)	
IIB	48 (51.0)	19 (46.3)	29 (54.7)	
IIIA	4 (4.3)	0 (0)	4 (7.5)	
IIIB	11 (11.7)	7 (17.1)	4 (7.5)	
Largest MRI tumour diameter, n (%)				
< 4 cm	28 (29.8)	13 (31.7)	15 (28.3)	NS
4 to 6 cm	27 (27.7)	12 (29.3)	14 (26.4)	
> 6 cm	15 (22.3)	4 (9.8)	11 (20.8)	
UK	24 (25.5)	10 (24.4)	14 (26.4)	
Lymph node involvement according to initial workup, n (%)				
Pelvic	32 (34.0)	13 (31.7)	19 (35.8)	NS
Para-aortic	0 (0)	0 (0)	(0)	
Initial workup, n (%)				
Pelvic MRI	87 (92.6)	40 (97.6)	47 (94.0)	NS
Cystoscopy	33 (35.1)	15 (37.5)	18 (36.7)	NS
PET-CT	31 (33.0)	11 (28.9)	20 (40.0)	NS
Initial haemoglobin, g/dL, median (IQR)	12.9 (11.9–14.2)	13.1 (12.3–14.2)	12.8 (11.5–13.9)	NS

Lymph node involvement is assessed according to the initial workup

Abbreviations: *CCRT* Concomitant Chemoradiotherapy, *IQR* Interquartile Range, *PS* Performance Status, *UK* Unknown, *EDI* European Deprivation Index, *FIGO* International Federation of Gynaecology and Obstetrics, *MRI* Magnetic Resonance Imaging, *PET-CT* Positron Emission Tomography-Computed Tomography

^a^Chi square test

### *Work-up and treatment* (Table 3)

The initial work-up included MRI for 92.6% of patients, cystoscopy for 35.1% and PET-CT for 33.0%. An optimal CCRT (i.e. concurrent chemoradiotherapy with at least five cycles of chemotherapy followed by a uterovaginal brachytherapy) was performed in 41 patients (43.6%). IMRT or VMAT were delivered to 37 patients (39.4%). Regarding patients treated in 2011–2014, IMRT or VMAT were performed in 85.2% of cases. The median pelvic dose was 46 Gy (IQR = 46–48), with 1.8–2.5 Gy per fraction. The median number of fractions was 23 (IQR = 23–25). Concurrent chemotherapy was delivered in 81 patients (86.2%), mainly with cisplatin (79.0%). The mean number of chemotherapy injections was 4 (IQR = 3–5). Brachytherapy was delivered to 73 patients (77.7%), mainly with PDR (*n* = 72, 76.6%). The mean brachytherapy dose was 25 Gy (IQR = 24–30). The median OTT was 56 days (IQR = 50–63).

**Table 3:** Treatment characteristics (*n* = 94)

Characteristics	Whole set of patients (*n* = 94)	Complete CCRT (*n* = 41)	Incomplete CCRT (*n* = 53)	*p-*value
Pelvic external radiation therapy, n (%)	94 (100)	41 (100)	53 (100)	NS
Total dose, median (IQR)	46 (46–48)	46 (46–48)	46 (46–48)	NS
Total dose boost, median (IQR)	55 (54–56)	56 (54–56)	56 (54–57)	NS
Fractions, median (IQR)	23 (23–25)	23 (23–25)	23 (23–25)	NS
Para aortic external radiation therapy, n (%)	3 (3.2)	1 (2.4)	2 (3.8)	NS
Radiotherapy technique				
3D–CRT, n (%)	57 (60.6)	26 (63.4)	31 (58.5)	NS
IMRT, VMAT, n (%)	37 (39.4)	15 (36.6)	22 (41.5)	
Concurrent chemotherapy, n (%)	81 (86.2)	41 (100)	40 (75.5)	< 0.01^a^
Number of cycles ≥ 4, n (%)	69 (73.4)	41 (100)	28 (52.8)	< 0.01^a^
Cisplatin, n (%)	64 (68.1)	34 (82.9)	30 (56.6)	< 0.01^b^
Carboplatin, n (%)	22 (23.4)	11 (26.8)	11 (20.8)	NS
Brachytherapy, n (%)	73 (77.7)	41 (100)	32 (60.4)	< 0.01^a^
PDR, n (%)	72 (76.6)	40 (97.6)	32 (60.4)	< 0.01^b^
HDR, n (%)	1 (1.1)	1 (2.4)	0 (0)	NS
Dose (Gy), median (IQR)	25 (24–30)	26 (24–30)	25 (24–30)	NS
OTT (days), median (IQR)	56 (50–63)	55 (50–64)	56 (53–62)	NS
Surgery, n (%)	77 (81.9)	35 (85.4)	42 (79.2)	NS
CHL, n (%)	55 (58.5)	27 (65.9)	28 (52.8)	NS
CHL and para aortic lymphadenectomy, n (%)	7 (7.4)	5 (12.2)	2 (3.8)	NS
Other, n (%)	15 (16)	2 (4.9)	11 (20.8)	< 0.03^a^
Time between brachytherapy and surgery (days), median (IQR)	42 (36–57)	42 (34–53)	45 (38–57)	NS

Lymph node involvement is assessed according to the initial workup

Abbreviations: *CCRT* Concomitant Chemoradiotherapy, *IQR* Interquartile Range, *3D–CRT* 3D Conformal Radiotherapy, *IMRT* Intensity Modulated Radiotherapy, *VMAT* Volumetric Modulated Arc Therapy, *PDR* Pulsed Dose Rate, *HDR* High Dose Rate, *Gy* Gray, *OTT* Overall Treatment Time, *CHL* Colpohysterectomy with bilateral adnexectomy and pelvic Lymphadenectomy

^a^Fisher test, ^b^Chi square test

Most patients underwent completion surgery (*n* = 77, 81.9%), mainly with CHL (*n* = 67, 71.3%). An additional para aortic lymphadenectomy was performed in 30 patients (31.9%): 23 before the chemoradiotherapy (24.5%), and 7 simultaneously with the pelvic surgery (7.4%). Para-aortic lymph node involvement was reported in 4 out of the 7 patients undergoing a post radiation lymphadenectomy.

### *Survival* (Fig. 1)

With a median follow-up of 43.8 months, the median OS time was 7.6 years. The median DSS time was 12.5 years. The 3-year and 5-year OS were 70.0% (95% CI = 61.1–82.2) and 61.9% (95% CI = 52.3–73.2) respectively. The 3-year and 5-year DSS were 73.8% (95%CI = 65.1–83.6) and 68.5% (95%CI = 59.2–79.2) respectively. The 5-year DFS was 56.7% (95% CI = 46.9–68.5). There was a trend toward decrease of OS for patients who had not received the standard care. Completion surgery was associated with a better OS.

**Fig. 1:**
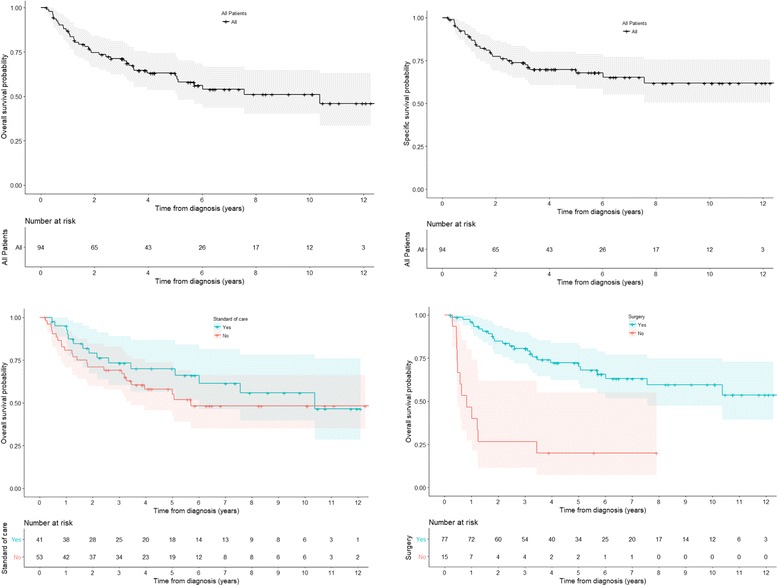
Survival curve are given with their 95% confident interval (95% CI); Five-year Overall Survival: 61.9% (95% CI = 52.3–73.2); Five-year Disease-Specific Survival: 68.5% (95%CI = 59.2–79.2); Median follow up: 43.9 months (Interquartile Range = 19.1–76.6); Mean follow up: 54.3 months

### *Prognostic factors of OS* (Table 4)

Performance status ≤ 1, uninvolved lymph nodes and concurrent chemotherapy were independent positive prognostic factors of OS in multivariate analysis. For patients with performance status ≤ 1, or without lymph nodes metastasis, there were not enough events to reach the median OS. Lower bounds of 95% CI were respectively 124.0 months and 73.1 months. When concurrent chemotherapy was performed, the median OS was 125 months.

**Table 4:** Prognostic factors of overall survival: univariate and multivariate analysis

Variables	Tested vs adverse criteria	Adverse criterion present median overall survival (months)	Adverse criterion absent median overall survival (months)	*p-*value (Log rank test)	Adjusted Hazard Ratio	95% CI	*p-*value (Cox model)
Age (years)	≥ 65 (vs < 65)	73.1	90.9	*p* = 0.361	–	–	–
PS	PS > 1 (vs ≤ 1)	48.6	> 124.0*	*p* < 0.001	2.86	1.41–5.79	*p* = 0.003
Histology	No SCC (vs SCC)	61.2	124.9	*p* = 0.262	–	–	–
Differentiation	Moderate or low (vs high)	73.1	90.9	*p* = 0.899	–	–	–
FIGO stage	III (vs IB2 and II)	37.4	90.9	*p* = 0.336	–	–	–
Pelvic lymph nodes involvement	Yes (vs no)	41.1	> 73.1*	*p* = 0.003	1.95	1.03–3.69	*p* = 0.040
MRI tumor size (mm)	≥ 50 (vs < 50)	32	> 124.9*	*p* = 0.006	–	–	–
Hemoglobin (g/dL)	< 11 (vs ≥ 11)	13.0	> 63.0*	*p* = 0.013	–	–	–
Radiotherapy	3D–CRT (vs IMRT or VMAT)	73	> 61.0*	*p* = 0.500			
Concurrent chemotherapy	No (vs yes)	17	125.0	*p* = 0.014	2.33	1.06–5.16	*p* = 0.035
Brachytherapy	No (vs yes)	37.4	124.9	*p* = 0.428	–	–	–
OTT (days)	> 55 (vs ≤ 55)	62.9	124.9	*p* = 0.428	–	–	–
Complete tumor sterilization	No (vs yes)	90.9	> 69.1*	*p* = 0.256	–	–	–

Abbreviations: *CI* Confident Interval, *SCC* Squamous Cell Carcinoma, *PS* Performance Status *FIGO* International Federation of Gynecology and Obstetrics, *MRI* Magnetic Resonance Imaging; *3DCRT* 3D Conformal Radiotherapy, *IMRT* Intensity Modulated Radiotherapy, *VMAT* Volumetric Modulated Arc Therapy, *OTT* Overall Treatment Time

Legend: Univariate analysis was performed using Log-rank test, multivariate analysis using Cox model

* = 95% CI lower bound (not enough event to reach median overall survival)

### Local control

Complete tumour sterilisation was achieved in 37 patients (55.2%). A partial response rate was achieved in 23 patients (34.3%). No association between tumour sterilisation and FIGO stage or OTT was found. At the end of follow-up, the local recurrence rate was 18.1%. The mean time to local recurrence was 14.2 months (IQR = 7.2–18.6).

## Discussion

This retrospective study highlighted that only a minority (43.6%) of LACC patients underwent the standard care. This was previously suggested in 15,194 American cervical cancer patients, with only 44.3% receiving the standard care [2]. Similar data was found in young LACC patients (< 65 years), with only 44% undergoing radio-chemotherapy for at least four concurrent cycles and brachytherapy [27]. Worse, only 25% completed the treatment in less than 56 days. In the present study, the OTT was longer than 56 days for half of patients. Although an OTT ≤ 56 days improves the pelvic control, it does not seem to impact anymore on OS and DSS since concurrent chemoradiation was developed [28, 29].

American studies had shown that high volume centres, higher density of radiation oncologists, academic centres, comprehensive community cancer centres, private insurance, higher income, and younger age were all associated with an increased likelihood of receiving standard care, whereas Black patients were less likely to receive standard care [2, 11, 27]. Adherence to standard care in high volume centres with high density of radiation oncologists probably reflects that access to the multidisciplinary resources needed (gynaecological oncologists, radiation oncologists expert in EBRT and brachytherapy, anaesthetists, operating suites, other radiation facilities equipped with a brachytherapy) and coordination are challenging. Territorial or socio-economic inequalities could not be accurately studied in this mono-centric study. Data from other European cancer care centres is needed.

We found that there was a trend towards decrease of adherence to standard care with age. However, the main characteristic associated with adherence to standard care was the performance status. This data suggests that deviation from the recommended treatment could result from treatment tailoring. Incidentally, overall survival was not significantly higher for patients receiving standard care than for others. Conversely, overall survival was significantly lower for patients who did not undergo a completion surgery, probably reflecting their frailty (contraindication to surgery) more than surgery benefit.

The fact that many publications only included patients receiving standard care widely restricts the external validity of researches [5, 8, 28]. This point raises serious concerns on outcome interpretation since it was demonstrated that deviations from standard care in multi-centre phase III clinical trials was associated with decreased survival [2, 28, 29]. Moore et al. analysed the treatment received by 1490 patients with a LACC, treated in Gynecologic Oncology Group (GOG) phase I-III trials [11]. Even in well-conducted clinical trials, the treatment significantly varied with age. Brachytherapy was reported to be performed less often in elderly patients, although it has been suggested it is safe among the oldest patients [11, 14, 21]. In the present study, brachytherapy was delivered in only three quarters of patients. Previous studies assessing real-life practice have shown similar data (55% to 88% in North America) [21, 30]. Yet, OS and PFS were proven to be increased by brachytherapy and survival rates of patients receiving brachytherapy were higher than patients treated with external boost [2, 20, 21]. Guidelines recommend delivering high dose rate (HDR) brachytherapy, planned with CT-scan or MRI [29, 31]. However, as it has been suggested that CT-scanning can overestimate the tumour width compared with MRI, MRI-based brachytherapy might allow a better dose-volume adaptation and dose escalation [32]. Consequently, MRI-based brachytherapy could give better local control, and fewer side effects [31–37]. It therefore seems to be an interesting option, even in poor condition patients, for which brachytherapy tolerance is sometimes questioned.

Patients > 65 years old represented less than 1% of the overall population in randomised clinical trials assessing concurrent cisplatin-based chemotherapy [17]. This result suggests that the standard care was not properly assessed in the geriatric population. The absence of a consensus concerning elderly-population-adapted treatments makes any therapeutic decision difficult. An onco-geriatric evaluation should therefore be performed before any treatment, in order to optimise the therapeutic strategy. In older people, comorbidities associated with cancer are known to induce poorer prognoses and lower adherence to standard care [12, 13]. In the present study, the Charlson combined age-comorbidity index revealed severe comorbidities in 26.6% of patients, at least partly explaining why a concurrent chemotherapy was only delivered in 86.0% of patients, with 68.1% receiving cisplatin. The RetroEMBRACE international cohort study reported that 76.5% of cervical cancer patients were prescribed chemotherapy between 1998 and 2012 [6]. The American National Cancer Database reported very similar results, with 74.7% in 2004–2012 [2]. However, chemotherapy is known to impact the OS. The Cochrane review reported a 6% improvement in 5-year OS with chemoradiotherapy, an 8% improvement in 5-year progression free survival (PFS), and a 9% improvement in 5-year local control [17]. The absolute benefit in OS and PFS was estimated at 12% (95% CI = 8–16) and 16% (95% CI = 13–19) respectively [18].

Age and comorbidities did not appear to impact EBRT treatment. In the present study, EBRT was always delivered, with a median pelvic dose of 46 Gy (IQR = 46–48). Technically advanced EBRT was used, often with IMRT or VMAT. IMRT is currently the recommended radiotherapy technique to treat LACC. It has been shown to preserve critical organs better than 3D–CRT, particularly bone marrow and bowels [38, 39]. It may enable the intensification of radiosensitisation or the addition of adjuvant treatment, particularly in cases with poor prognostic factors. One of the most important poor prognostic factors is lymph node involvement, which was reported in 34.1% of participants in this study. It was statistically associated with lower OS, as described in the GOG phase I-III trials analysis [11]. When stratified by FIGO stage, the hazard ratio associated with positive lymph nodes is estimated at 3.3 (95% CI 2.8–4.0) in cervical cancer patients [9]. The presence, in addition to the level, of nodal spread were described as prognostic factors of OS [5]. This strong association between lymph node involvement and OS could therefore suggest identifying LACC with and without lymph nodes involvement, and to consider different therapeutic strategies. Many clinical trials are assessing treatment intensification, with immunotherapy, target therapy or antiviral treatment addition (NCT03298893, NCT02705612, NCT02501278, NCT01217177, NCT00023660, NCT00369122, etc), concomitant chemotherapy intensification (NCT01561586, NCT00292955, NCT00548821, etc), or adjuvant treatment (NCT02036164, NCT02853604, etc). Other clinical trials are assessing new treatment strategies, such as alternative medical treatments or improvements in coordination of care. The European Organisation for Research and Treatment of Cancer (EORTC) is comparing the effectiveness of chemotherapy followed by surgery, versus CCRT in patients with stage IB or II cervical cancer (EORTC 55994, NCT00039338). This strategy could be an interesting alternative to CCRT, which seems difficult to observe in a real-world setting.

## Conclusions

OS and OSS were previously often estimated in patients receiving the standard care. However, previous American real-life studies revealed that less than 50% of the patients underwent it. The present study confirms this result in a French Cancer Care Centre, reflecting how often heterogeneous patient and disease characteristics required treatment adaptation, and how often physician communication and coordination can result in difficulties in providing multiple components of cervical cancer treatment. Selected patients in clinical trials do not seem to accurately reflect the characteristics of the real-world population. The standard care of frail patients is still to be defined, but involvement of oncogeriatrics may already be enhancing elderly patients’ outcomes. For fit patients with poor prognostic factors, such as lymph node involvement, escalation therapy could be assessed in clinical trials. More data about treatment and outcomes in a real-world setting is required in order to optimise clinical trials and daily clinical practice.

## Abbreviations

3D–CRT: 3D–Conformal radiotherapy
AIC: Akaike Information Criterion
AUC: Area under the curve
CBCT: Cone-beam computed tomographic
CCRT: Concomitant chemoradiotherapy
CHL: Colpohysterectomy with bilateral adnexectomy and pelvic Lymphadenectomy
CI: Confident interval
CT-scan: Computed Tomography-scan
CTV: Clinical target volume
DFS: Disease-free survival
DSS: Disease-specific survival
EBRT: External beam radiation therapy
EDI: European Deprivation Index
EORTC: European Organisation for Research and Treatment of Cancer
FIGO: International Federation of Gynecology and Obstetrics
GOG: Gynecologic Oncology Group
GTV: Gross Tumor Volume
Gy: Gray
HDR: High dose rate
HPV: Human papillomavirus
ICRU: International Commission on Radiation Units and measurements
IMRT: Intensity modulated radiotherapy
IQR: Interquartile range
LACC: Locally advanced cervical cancer
MRI: Magnetic resonance imaging
OS: Overall survival
OTT: Overall treatment time
PDR: Pulsed-dose rate
PET-CT: Positron Emission Tomography-Computed Tomography
PFS: Progression free survival
PTV: Planning target volume
VMAT: Volumetric modulated arc therapy
