# Correction to: From IB2 to IIIB locally advanced cervical cancers: report of a ten-year experience

**DOI:** 10.1186/s13014-018-0999-9

**Published:** 2018-03-23

**Authors:** Sophie Espenel, Max-Adrien Garcia, Jane-Chloé Trone, Elodie Guillaume, Annabelle Harris, Amel Rehailia-Blanchard, Ming Yuan He, Sarra Ouni, Alexis Vallard, Chloé Rancoule, Majed Ben Mrad, Céline Chauleur, Guy De Laroche, Jean-Baptiste Guy, Pablo Moreno-Acosta, Nicolas Magné

**Affiliations:** 1 Radiotherapy Department, Lucien Neuwirth Cancer Institute, 108 bis avenue Albert Raimond, BP60008, 42271 Saint-Priest-en-Jarez cedex, France; 2 Public Health Department, Lucien Neuwirth Cancer Institute, 108 bis avenue Albert Raimond, BP60008, 42271 Saint-Priest-en-Jarez cedex, France; 3 Chelsea and Westminster Hospital, 369 Fulham Road, London, SW10 9NH UK; 4 Obstetrics and Gynecology Department, Saint Etienne University Hospital Medical Center, avenue Albert Raimond, BP60008, 42271 Saint-Priest-en-Jarez cedex, France; 5 Research Group in Radiobiology Clinical, Molecular and Cellular, National Cancer Institute, Bogotá, Colombia; 6 Research Group in Cancer Biology, National Cancer Institute, Bogotá, Colombia

## Correction

In the original publication [1] one author name was spelled incorrect. The corrected version can be found in this Erratum. The original article was updated to rectify these errors.

Incorrect author name:

Céline Chaleur

Correct author name:

Céline Chauleur

## References

[1] Espenel S, Garcia MA, Trone JC, Guillaume E, Harris A, Rehailia-Blanchard A, He MY, Ouni S, Vallard A, Rancoule C, Ben Mrad M, Chaleur C, De Laroche G, Guy JB, Moreno-Acosta P, Magné N (2018). From IB2 to IIIB locally advanced cervical cancers: report of a ten-year experience. Radiat Oncol.

